# Commensal colonization reduces *Pseudomonas aeruginosa* burden and subsequent airway damage

**DOI:** 10.3389/fcimb.2023.1144157

**Published:** 2023-05-25

**Authors:** Sara N. Stoner, Joshua J. Baty, Lea Novak, Jessica A. Scoffield

**Affiliations:** Department of Microbiology, University of Alabama at Birmingham, Birmingham, AL, United States

**Keywords:** cystic fibrosis, *Pseudomonas aeruginosa*, *Streptococcus salivarius*, oral commensal bacteria, polymicrobial, streptococci, commensal

## Abstract

*Pseudomonas aeruginosa* dominates the complex polymicrobial cystic fibrosis (CF) airway and is a leading cause of death in persons with CF. Interestingly, oral streptococcal colonization has been associated with stable CF lung function. The most abundant streptococcal species found in stable patients, *Streptococcus salivarius*, has been shown to downregulate pro-inflammatory cytokines in multiple colonization models. However, no studies have demonstrated how *S. salivarius* potentially improves lung function. Our lab previously demonstrated that the *P. aeruginosa* exopolysaccharide Psl promotes *S. salivarius* biofilm formation *in vitro*, suggesting a possible mechanism by which *S. salivarius* is incorporated into the CF airway microbial community. In this study, we demonstrate that co-infection of rats leads to enhanced *S. salivarius* colonization and reduced *P. aeruginosa* colonization. Histological scores for tissue inflammation and damage are lower in dual-infected rats compared to *P. aeruginosa* infected rats. Additionally, pro-inflammatory cytokines IL-1β, IL-6, CXCL2, and TNF-α are downregulated during co-infection compared to *P. aeruginosa* single-infection. Lastly, RNA sequencing of cultures grown in synthetic CF sputum revealed that *P. aeruginosa* glucose metabolism genes are downregulated in the presence of *S. salivarius*, suggesting a potential alteration in *P. aeruginosa* fitness during co-culture. Overall, our data support a model in which *S. salivarius* colonization is promoted during co-infection with *P. aeruginosa*, whereas *P. aeruginosa* airway bacterial burden is reduced, leading to an attenuated host inflammatory response.

## Introduction

*Pseudomonas aeruginosa* causes multi-drug resistant infections in persons with cystic fibrosis (CF), which lead to chronic inflammation and a subsequent decline in lung function (Bhagirath et al., 2016; Martinez-García et al., 2021). *P. aeruginosa* infection causes significant neutrophil influx and pro-inflammatory cytokine release, which is ineffective at clearing *P. aeruginosa* from the CF lung (Hayes et al., 2011). Additionally, CFTR dysfunction has been shown to induce elevated inflammatory cytokines and neutrophilic response in the absence of bacterial infection (Roesch et al., 2018). This combination of dysregulated inflammatory responses in the absence of functioning CFTR and further inflammatory induction by *P. aeruginosa* infection can lead to lung tissue damage and decline in lung function (Roesch et al., 2018).

Multiple studies have suggested that *Streptococcus* species are associated with stable CF lung function (Filkins et al., 2012; Coburn et al., 2015). The most prevalent *Streptococcus* species found in stable persons with CF was *Streptococcus salivarius*, a commensal commonly found in the oral cavity (Filkins et al., 2012). *S. salivarius* has been shown to downregulate inflammatory cytokines in multiple cell models of colonization (Cosseau et al., 2008; Guglielmetti et al., 2010; Kaci et al., 2011; Kaci et al., 2014). Additionally, *S. salivarius* can inhibit inflammatory cytokine release that is stimulated by bacterial pathogens including *P. aeruginosa* in cell models (Cosseau et al., 2008; MacDonald et al., 2021). Further, the oral commensal *Streptococcus mitis* was shown to reduce *P. aeruginosa*-induced inflammation in a murine infection model (Tony-Odigie et al., 2022).

Oral commensals including *S. salivarius* have not only been shown to downregulate host inflammatory pathways, but also inhibit pathogen growth and colonization. For example, *S. salivarius* has also been shown to inhibit colonization of *Streptococcus pneumoniae* on epithelial cells (Manning et al., 2016). Additionally, *S. salivarius* inhibits growth of the oropharyngeal pathogen *Streptococcus pyogenes* via bacteriocin production (Upton et al., 2001). Although *S. salivarius* has been shown to downregulate host pro-inflammatory pathways and inhibit growth of specific respiratory pathogens, no studies have examined how *S. salivarius* impacts *P. aeruginosa* pathogenesis during airway infection.

We previously demonstrated that *S. salivarius* biofilm formation and colonization of *Drosophila melanogaster* is promoted by the *P. aeruginosa* exopolysaccharide Psl, serving as a possible mechanism by which *S. salivarius* incorporates into the CF airway microbial community (Stoner et al., 2022). In an effort to understand how these interspecies interactions affect lung function during *P. aeruginosa* infection, we utilized a rat co-infection model. We found that co-inoculation with *S. salivarius* decreased *P. aeruginosa* colonization. Pro-inflammatory cytokines IL-1α, IL-1β, IL6, TNF-α, and CXCL-2 were downregulated in co-infected groups compared to animals infected with *P. aeruginosa* alone. Additionally, histological scores were lower on average in dual-infected animals compared to *P. aeruginosa*-infected animals. Finally, *P. aeruginosa* glucose metabolism genes were downregulated in the presence of *S. salivarius* in synthetic CF sputum, suggesting a potential alteration in *P. aeruginosa* fitness during dual infection in the airway. Overall, our study supports a mechanism by which *S. salivarius* decreases *P. aeruginosa* bacterial burden in the rat lung, which leads to a decrease in airway inflammation and tissue damage.

## Results

### *S. salivarius* colonization is promoted and *P. aeruginosa* colonization is inhibited in co-infected rats

**Figure 1:**
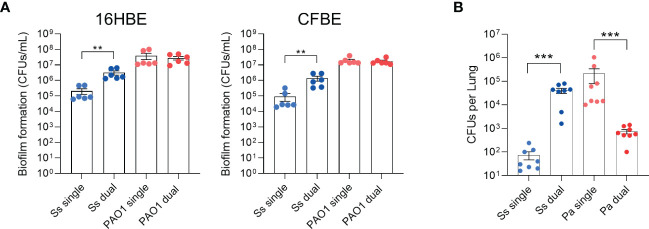
*S. salivarius* colonization is promoted while *P. aeruginosa* colonization is inhibited in a dual-infection rat model. **(A)** 16HBE and CFBEs were infected with Ss and/or PAO1 for 6 hours (n=3 biological, 2 technical replicates). **(B)** Rats were intranasally inoculated with overnight cultures of Ss (~10^7^ CFU) and/or PAO1 (~10^8^ CFU), or PBS. After 16 hours, CFUs from lung homogenates were quantified. Mann-Whitney test. Error bars indicate standard error of the mean, n = 8. **P<0.01, ***P<0.001.

We previously reported that the non-mucoid *P. aeruginosa* strain PAO1 promotes both *S. salivarius* biofilm formation *in vitro* as well as colonization in a *Drosophila* oral infection model (Stoner et al., 2022). To determine whether *P. aeruginosa* promotes *S. salivarius* colonization in the context of the CF lung, we infected wildtype bronchial epithelial cells (16HBE) and CFTR KO bronchial epithelial cells (CFBE) with PAO1 and *S. salivarius* strain K12 and quantified adherent CFUs after 6 hours (**Figure 1A**). *S. salivarius* colonization increased significantly in the presence of *P. aeruginosa*, while no change in *P. aeruginosa* colonization was observed. Next, to determine whether *S. salivarius* is promoted and impacts *P. aeruginosa* pathogenesis in a mammalian model, we co-infected Sprague Dawley rats simultaneously with *P. aeruginosa* (~10^8^ CFU) and *S. salivarius* (~10^7^ CFU). Consistent with our previous findings, *S. salivarius* colonization significantly increased in the presence of *P. aeruginosa* (**Figure 1B**). However, contrary to our previous data showing no effect on *P. aeruginosa* growth by *S. salivarius in vitro*, we observed that *P. aeruginosa* colonization of the rat airway is inhibited in the presence of *S. salivarius*. These data indicate that this oral commensal may provide protection in the CF airway by restricting *P. aeruginosa* colonization.

### Lung tissue inflammation is attenuated in dual-infected rats

**Figure 2:**
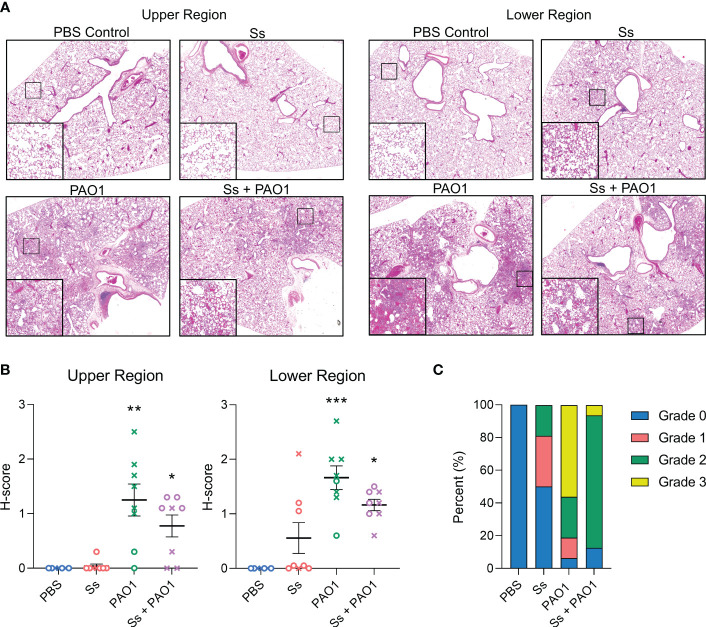
Lung inflammation is attenuated in dual-infected rats. Sprague Dawley rats were intranasally inoculated with PAO1 (~10^8^ CFU) and/or Ss (10^7^ CFU) and euthanized 16 hours post-infection. **(A)** Hematoxylin and eosin staining of upper and lower lung sections (100x magnification). **(B)**. Severity of inflammation was graded in a blinded fashion by a board-certified pathologist (L.N.). **(C)** Parts of a whole graph showing the highest maximum grades of individual rats in each infection group. Error bars indicate standard error of the mean, n = 7-8, X = female, O = male. Kruskal Wallis with Dunn’s multiple comparisons test. *P<0.05, **P<0.01, ***P<0.001.

Since *P. aeruginosa* colonization was inhibited in the presence of *S. salivarius*, we examined whether tissue damage was also reduced in the presence of *S. salivarius* during *P. aeruginosa* infection (**Figure 2A**). Upper and lower regions of the left lung were scored in a blind fashion for severity of inflammation. On average, *S. salivarius* and *P. aeruginosa* single and dual infections induced more inflammation in the lower airway compared to the upper airway (**Figure 2B**). *S. salivarius* alone induced mild neutrophil influx to the lungs with an average histopathological score of 0.037 for the upper region and 0.55 for the lower region. Overall, *P. aeruginosa* single infection induced more airway inflammation than dual infection with *S. salivarius* and *P. aeruginosa*. In the upper region of the airway, *P. aeruginosa* single-infected lungs had an average histological score (H-score) of 1.25, while dual infected lungs had an H-score of 0.76. In the lower regions of the airway, *P. aeruginosa*-infected lungs received an average H-score of 1.66, while dual-infected lungs received an average H-score of 1.16 (**Figure 2B**). All uninfected control rats and half of *S. salivarius*-infected rats had a maximum histology grade of 0. Over half of *P. aeruginosa*-infected rats had areas of inflammation with a maximum grade of 3, which is marked by significant neutrophilic influx and irreversible damage to alveolar walls. Notably, over 80 percent of dual-infected rats had areas of inflammation with a maximum grade of 2, indicating significant neutrophil influx and preservation of alveolar wall structure (**Figure 2C**). Taken together, these data suggest that dual-infected rats are better protected from permanent alveolar wall damage during *P. aeruginosa* infection.

### Pro-inflammatory cytokines are downregulated in dual-infected rats

**Figure 3:**
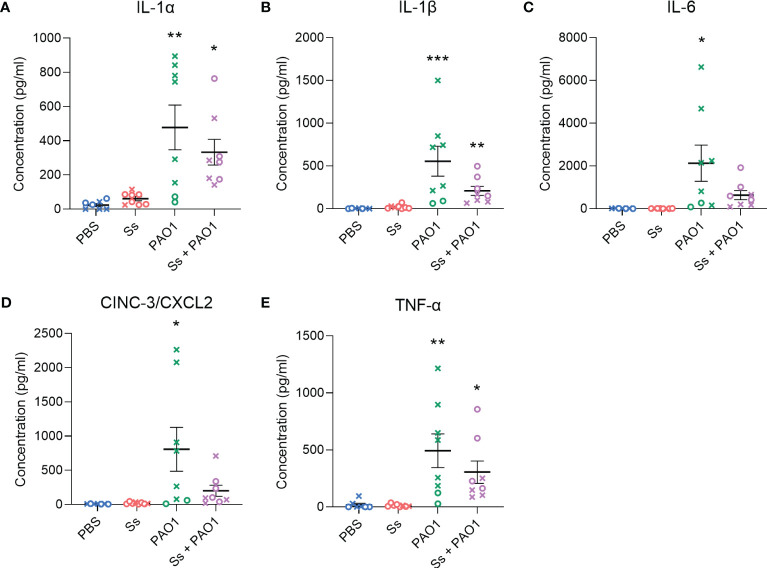
Inflammatory cytokines are downregulated in dual-infected rats. **(A–E)** Cytokine analysis was performed on BAL supernatant of rats infected with Ss and/or PAO1, or PBS for 16 hours. Error bars indicate standard error of the mean, n = 7-8, X = female, O = male. One-way ANOVA with Šίdák’s multiple comparisons test for parametric data, or Kruskal-Wallis with Dunn’s multiple comparisons test for nonparametric data. *P<0.05, **P<0.01, ***P<0.001.

To further characterize the inflammatory response to *P. aeruginosa* in the presence of *S. salivarius*, inflammatory cytokines and chemokines that play a role in innate immunity were measured, including IL-1α, IL-1β, IL-6, CXCL2, and TNF-α (**Figure 3**). *P. aeruginosa* single-infection elicited a robust response in all cytokines and chemokines, while *S. salivarius* single-infection did not elicit a significant response compared to un-infected controls. While not statistically significant, all cytokine levels were substantially lower in dual-infected rats compared to *P. aeruginosa*-infected rats. Notably, IL-6 and CXCL2 levels were not significantly different in dual-infected rats compared to uninfected controls (**Figures 3C, D**). No significant changes in IL-10 levels were observed between all four infection groups (**Figure S1A**). IFN-γ was induced in both *P. aeruginosa*-infected and dual-infected rats (**Figure S1B**).

### Neutrophil recruitment and activity are not affected by *S. salivarius* during *P. aeruginosa* infection

**Figure 4:**
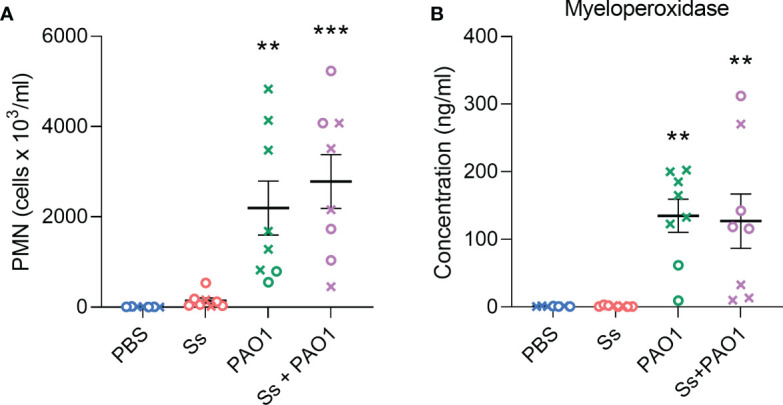
Neutrophil recruitment and activity are not altered by *S. salivarius* presence during *P. aeruginosa* infection. **(A)** Quantification of absolute neutrophil counts in BAL samples after intranasal inoculation with Ss and/or PAO1, or PBS. Kruskal Wallis with Dunn’s multiple comparisons test. **(B)** Quantification of myeloperoxidase in BAL supernatant. One-way ANOVA with Šίdák’s multiple comparisons test. Error bars indicate standard error of the mean, n = 7-8, X = female, O = male. **P<0.01, ***P<0.001.

We investigated whether neutrophil recruitment or activity was altered by the presence of *S. salivarius* during *P. aeruginosa* infection (**Figure 4A**). When measuring absolute counts of neutrophils, *S. salivarius* alone did not stimulate significant neutrophil recruitment compared to uninfected animals. Both *P. aeruginosa* infection and dual infection elicited neutrophil recruitment to the lungs, however, there was no significant difference between the two groups. Myeloperoxidase levels were then measured as a function of neutrophil activation (**Figure 4B**). *S. salivarius* alone did not induce significant neutrophil activation compared to uninfected controls. Both *P. aeruginosa* infection and dual infection induced myeloperoxidase release, but the presence of *S. salivarius* during *P. aeruginosa* infection did not affect myeloperoxidase levels.

### *P. aeruginosa* sugar metabolism genes are downregulated in the presence of *S. salivarius*

**Figure 5:**
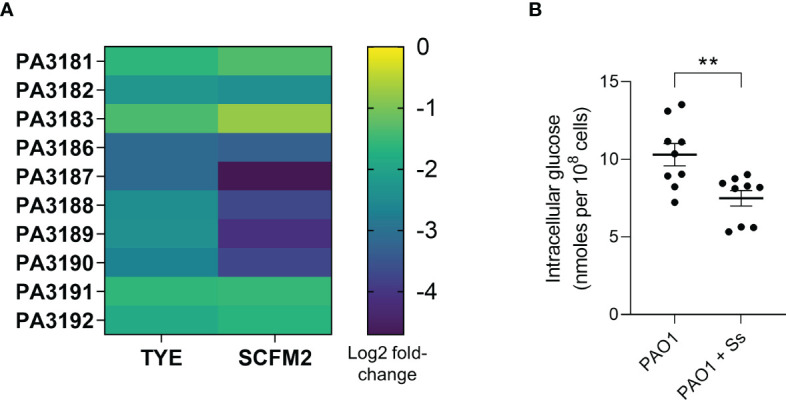
*P. aeruginosa* sugar metabolism genes are downregulated in the presence of *S. salivarius*. **(A)** Heat map demonstrating differential expression of ten PAO1 sugar metabolism genes in the presence of *S. salivarius*- PA3181 (2-keto-3-deoxy-6-phosphogluconate aldolase EdaA), PA3182 (6-phosphogluconolactonase Pgl), PA3183 (glucose-6-phosphate 1-dehydrogenase Zwf), PA3186 (glucose outer membrane porin OprB), PA3187 (ATP binding component of ABC sugar transporter GltK), PA3188 (permease of ABC sugar transporter GltG), PA3189 (permease of ABC sugar transporter GltF), PA3190 (binding protein component of ABC sugar transporter), PA3191 (glucose transport sensor GtrS), and PA3192 (two-component response regulator GltR). **(B)** PAO1 intracellular glucose levels in the presence or absence of Ss. Mann-Whitney test. Error bars indicate standard error of the mean (n=3 biological, 3 technical replicates). **P<0.01.

To understand how *S. salivarius* may inhibit *P. aeruginosa* growth in the airways, we measured the transcriptomic response of *P. aeruginosa* genes in the presence and absence of *S. salivarius* (**Figure 5A**). *S. salivarius* and *P. aeruginosa* biofilm samples were cultured *in vitro* in either tryptic soy broth medium (TSBYE) or a synthetic cystic fibrosis sputum medium (SCFM2), which accurately mimics the nutritional environment of the CF airway (Turner et al., 2015). Additionally, the fitness of *P. aeruginosa* in SCFM2 is comparable to that of *P. aeruginosa* in CF sputum samples recovered from the lung (Turner et al., 2015). RNA sequencing revealed the downregulation of ten genes involved in *P. aeruginosa* glucose metabolism in the presence of *S. salivarius*. Genes PA3186 (*oprB*), PA3187 (*gltK*), PA3188 (*gltG*), PA3189 (*gltF*), and PA3190 are involved in glucose uptake. PA3181 (*edaA*), PA3182 (*pgl*), and PA3183 (*zwf*) are involved in the catabolic conversion of glucose to pyruvate. PA3191 (*gtrS*) and PA3192 (*gltR*) comprise a two-component system that regulates expression of specific genes involved in glucose metabolism (Suzuki and Wood, 1980; Ma et al., 1998; Hager et al., 2000; Daddaoua et al., 2014). To confirm our RNA sequencing results that suggested *S. salivarius* reduces the ability of *P. aeruginosa* to uptake glucose, we measured intracellular glucose levels in *P. aeruginosa* during co-culture with *S. salivarius*. *P. aeruginosa* was cultured in SCFM2 in the presence or absence of *S. salivarius*. *S. salivarius* was separated from *P. aeruginosa* by a 0.4μM transwell insert to properly isolate *P. aeruginosa* for glucose measurements after culturing. After 6 hours, *P. aeruginosa* intracellular glucose levels were significantly lower in the presence of *S. salivarius* compared to *P. aeruginosa* single-species cultures (**Figure 5B**). Overall, our data suggests that *S. salivarius* may interfere with the ability of *P. aeruginosa* to efficiently catabolize nutrients in the CF airway.

## Discussion

*P. aeruginosa* causes airway infections in persons with CF that lead to chronic inflammation and a decline in lung function. There is a growing body of literature demonstrating the effects of interspecies interactions on the behavior and virulence of *P. aeruginosa*, which may have a significant impact on clinical outcomes (Al-Wrafy et al., 2023). Remarkably, oral commensal streptococci have been correlated with improved lung function in the CF airway (Filkins et al., 2012; Cuthbertson et al., 2020). Additionally, a decrease in prevalence of streptococci has been associated with a decline in lung function (Coburn et al., 2015). Therefore, it is important to understand how members of the airway microbial community may prevent airway inflammation and tissue damage. In this study, we examined the role of *S. salivarius*, the most prominent commensal linked to stable CF lung function, on *P. aeruginosa* pathogenesis in a cell culture and rat airway model. Our study reveals that *P. aeruginosa* promotes *S. salivarius* colonization in both wildtype and CF bronchial epithelial cells, and in a rat model of infection. Interestingly, *S. salivarius* reduced *P. aeruginosa* burden in the rat model, in addition to key pro-inflammatory markers and tissue damage. Moreover, transcriptomic analysis of dual cultures grown in SCFM2 indicated that the oral commensal downregulated genes that are important for *P. aeruginosa* to metabolize glucose, which could potentially hinder *P. aeruginosa’s* ability to compete for and metabolize nutrients in the polymicrobial airway. In summary, our study reveals diverse mechanisms by which *S. salivarius* may provide protection against *P. aeruginosa* during CF airway infection. Oral commensals including *S. salivarius* have previously been shown to downregulate inflammatory responses in cell culture and murine infection models (Cosseau et al., 2008; Guglielmetti et al., 2010; Kaci et al., 2011; Kaci et al., 2014; Tony-Odigie et al., 2022). Because *P. aeruginosa* bacterial burden is reduced during co-infection with *S. salivarius*, we cannot definitively conclude that the decrease in tissue inflammation and inflammatory cytokine release during co-infection is due to direct downregulation by *S. salivarius*. Additionally, the anti-inflammatory cytokine IL-10 was not significantly induced during *S. salivarius* single-infection or during dual infection. IL-10 is known to inhibit expression of inflammatory cytokines, including IL-1, IL-6, and TNF (Mosser and Zhang, 2008). These findings suggest that the observed decrease in some inflammatory cytokines during dual infection is not caused by an anti-inflammatory response induced by *S. salivarius*, but rather by a decrease in *P. aeruginosa* bacterial burden.

We demonstrated that the presence of *S. salivarius* decreases *P. aeruginosa* airway bacteria burden. We did not observe any increases in immune cell recruitment or activity during co-infection that would suggest that the immune system clears *P. aeruginosa* from the airway more efficiently in the presence of *S. salivarius*. Studies have shown that *S. salivarius* directly inhibits *S. pneumoniae* adhesion to pharyngeal cells (Manning et al., 2016). Therefore, *S. salivarius* may have the capacity to inhibit *P. aeruginosa* adherence to airway cells. Alternatively, the observed decrease in *P. aeruginosa* colonization may be due to changes in *P. aeruginosa* gene expression profile and behavior in the presence of *S. salivarius*. Ten genes involved in *P. aeruginosa* glucose uptake and metabolism were downregulated in the presence of *S. salivarius*. Additionally, *P. aeruginosa* intracellular glucose levels significantly decreased in the presence of *S. salivarius*. Previous work demonstrates that elevated glucose concentrations in the airway surface liquid (ASL) lead to increased *P. aeruginosa* bacterial burden (Garnett et al., 2013). Further, deletion of *P. aeruginosa* genes involved in glucose uptake have been shown to attenuate virulence in a *Galleria mellonella* model of infection (Raneri et al., 2018). Taken together, previous literature suggests that downregulation of *P. aeruginosa* glucose metabolism genes in the presence of *S. salivarius* may lead to reduced colonization efficiency and virulence. However, further experiments will need to be performed to confirm that decreased glucose uptake in the presence of *S. salivarius* leads to a decrease in *P. aeruginosa* colonization and fitness. While glucose metabolism genes were downregulated in the presence of *S. salivarius* in both TSBYE and SCFM2, we observed a more drastic decrease in SCFM2. This may be due to lower glucose availability in SCFM2 (13.8μM glucose) compared to TSBYE (3mM glucose) (Turner et al., 2015). Additionally, two downregulated genes identified via RNA sequencing comprise a two-component system- *gtrS*, a glucose transport sensor, and *gltR*, a two-component response regulator. GtrS has been shown to modulate colonization and dissemination in a murine infection model through modulation of the type III secretion system (T3SS) (O'Callaghan et al., 2012). Further studies are warranted to better understand how downregulation of *gltR* and *gtrS*, and glucose metabolism genes in general, affect *P. aeruginosa* fitness during infection, in addition to nutritional competition with airway microbiota.

*S. salivarius* colonization was significantly increased in the presence of *P. aeruginosa* in our bronchial epithelial cell and rat models of infection. We previously demonstrated that the exopolysaccharide Psl produced by *P. aeruginosa* strain PAO1 promoted *S. salivarius* biofilm formation *in vitro* and colonization in a *Drosophila melanogaster* model of infection (Stoner et al., 2022). This phenotype is not exclusive to *S. salivarius*; our lab previously demonstrated that biofilm formation of *Streptococcus parasanguinis*, another oral commensal, is promoted by the exopolysaccharide alginate produced by *P. aeruginosa* (Scoffield et al., 2017). Additionally, Psl is expressed by *P. aeruginosa* in the CF airway and is important for aggregate formation and persistence in the CF lung (Jennings et al., 2021; Morris et al., 2022). Therefore, the promotion of *S. salivarius* colonization that we observed on bronchial epithelial cells and in rat airways may be due to interactions between *S. salivarius* and Psl produced by *P. aeruginosa*. Additional research is needed to uncover the role of Psl in enhanced *S. salivarius* airway colonization and whether this increase in *S. salivarius* colonization plays a role in inhibition of *P. aeruginosa* during dual infection.

We observed that the highest inflammatory cytokine responses from *P. aeruginosa* infection were in female rats. This is supportive of clinical data that demonstrates females with CF have worse pulmonary exacerbation outcomes during *P. aeruginosa* infection than men (Montemayor et al., 2021). Additionally, adult women have been shown to mount stronger innate and adaptive immune responses than men (Klein and Flanagan, 2016). Research also suggests that estrogen increases *P. aeruginosa* pyocyanin production and swarming motility, which could lead to more severe *P. aeruginosa* infections in women (Tyrrell and Harvey, 2020). Further research and a larger sample size is needed to determine whether sex is related to altered clinical outcomes in a rat *P. aeruginosa* infection model.

While our findings help us better understand the impact of polymicrobial communities on *P. aeruginosa* airway infections, there are limitations in this study that warrant further discussion. The *P. aeruginosa* strain used in this co-infection model, PAO1, is a lab-adapted *P. aeruginosa* strain. Further studies with acute CF isolates of *P. aeruginosa* that are phenotypically similar to PAO1 are needed to confirm whether clinical isolates that more accurately mimic early *P. aeruginosa* infection in the CF airway recapitulate the same phenotype observed during dual infection with *S. salivarius*. Another limitation is that gene expression profiling of *P. aeruginosa* in the presence and absence of *S. salivarius* was performed on *in vitro* cultures grown in SCFM2. The gene expression profile of *P. aeruginosa* cultured in SCFM2 has been previously shown to closely mimic the expression profile of *P. aeruginosa* during infection of the CF airway (Turner et al., 2015). However, transcriptomic profiling of *P. aeruginosa* taken from rat lungs during single and dual infection may further inform about the impact of *S. salivarius* on *P. aeruginosa* behavior in the airway.

In summary, co-infection with *P. aeruginosa* and the oral commensal *S. salivarius* leads to reduced *P. aeruginosa* airway bacterial burden and, subsequently, reduced inflammation and tissue damage in the lungs. Additionally, the presence of *S. salivarius* may alter *P. aeruginosa* fitness and the ability to colonize the host via downregulation of *P. aeruginosa* glucose metabolism genes. This study highlights the potential of commensal streptococci to modulate CF airway disease caused by *P. aeruginosa* infection. Further studies will uncover specific mechanisms by which oral commensals, including *S. salivarius*, inhibit *P. aeruginosa* colonization and pathogenesis, which may lead to improved therapeutic strategies.

## Materials and methods

### Bacterial strains, cell lines, and growth conditions

Strains *S. salivarius* K12 and *P. aeruginosa* PAO1 were used in this study. *S. salivarius* was grown on Todd-Hewitt Broth (Cuthbertson et al., 2020) agar (Becton Dickinson) and cultured statically at 37 °C in 5% CO_2_ in THB. *P. aeruginosa* was grown on Pseudomonas Isolation Agar (PIA; Becton Dickinson) and cultured in Luria broth (LB; Fisher) and incubated while shaking (250 rpm) at 37 °C. Biofilm assays were performed on the immortalized wildtype human bronchial epithelial cell line 16HBE and the immortalized ΔF508/ΔF508 CF bronchial epithelial cell line CFBE41o- (Cozens et al., 1994). Cells were maintained with minimal essential medium (MEM) supplemented with 10% fetal bovine serum. Cells were polarized by seeding at a density of 5x10^5^ on the apical surface of transwell filters and growing at 37°C for 7 days before removing the apical media and growing for 7 days at air-liquid interface.

### Cell infection assays

*P. aeruginosa* and *S. salivarius* overnight cultures were normalized to OD_600_ 0.5 in minimal essential medium (MEM) supplemented with 5% L-glutamine. Cells were infected with normalized cultures that were diluted 1:25 in MEM and incubated for 1 hour at 37°C with 5% CO_2_. After 1 hour, inoculum was removed from cells, centrifuged at 13,000 rpm for 2 minutes to remove planktonic bacteria, and added back to cells. L-arginine was then added to inoculum for final concentration of 0.4% to encourage biofilm growth. Cells were then further incubated for 5 hours at 37°C. To quantify viable adherent bacteria, cells were washed with MEM, then treated with 0.1% Triton X-100 for 15 minutes to remove cells from transwells. Cells were then vortexed for 3 minutes, diluted, and plated on THB agar plates for bacterial counts.

### Rat model of respiratory infection

Sprague Dawley rats (8 weeks of age) were obtained from Taconic Biosciences (Albany NY). For single infections, rats were inoculated intranasally with 300ul *P. aeruginosa* (~10^8^ CFU) or *S. salivarius* (~10^7^ CFU) resuspended in phosphate buffered saline (PBS). For dual infections, rats were inoculated intranasally with 300ul PBS containing *P. aeruginosa* (~10^8^ CFU) and *S. salivarius* (~10^7^ CFU). Lungs were flushed with 4ml sterile PBS. Samples were stored on ice until processing, then centrifuged at 1500 rpm for 5 minutes. Supernatant was removed and stored at -80°C for future cytokine analysis, and pelleted cells were used for differential cell counting. The right lung of each rat was placed in 1ml sterile PBS, homogenized, and plated on Todd Hewitt Broth agar plates for viable bacterial cell quantification. The left lung of each rat was inflated with 10% neutral-buffered formalin and stored at 4°C for histological analysis. All rat infection protocols were approved by the University of Alabama at Birmingham (UAB) Institutional Animal Care and Use Committees (IACUC protocol 21546).

### Cytokine analysis and differential cell counting

BALF sample supernatants were used for cytokine quantification. CXCL2, IL-6, MPO, and TNF-α were measured using the CXCL2, IL-6, and TNF-α Quantikine ELISA kits, respectively (R&D Systems). IL-1α was measured using the Rat IL-1α ELISA kit (Novus Biologicals). IL-1β was measured using the Rat IL-1β ELISA kit (Invitrogen). Myeloperoxidase was measured using the Rat Myeloperoxidase ELISA kit (Abcam). For differential cell counts, BALF cell pellets were resuspended in 500μL PBS and collected via cytospin at 600 rpm for 10 minutes (Cytospin 4, Thermo Scientific). Cells were stained using the Kwik Diff Stain Kit (Thermo Scientific), and differential counting was performed on the EVOS FL Cell Imaging System. Counts from three representative areas were performed for each sample.

### Histological analysis

Left lungs were stored in 10% NBF at 4°C until processing. For each lung, three upper region sections and three lower regions were sent to the UAB Pathology Core Research Laboratory for paraffin tissue embedding, sectioning, and hematoxylin and eosin (H&E) staining. Imaging was performed using a Cytation 5 microscope at 100x magnification (Agilent BioTek). Semiquantitative analysis of lung sections was performed by a board-certified surgical pathologist (L.N.). Histological scoring was based primarily on neutrophilic influx and alveolar wall preservation. Severity was rated on a scale of 0 to 3, where 0 represents no inflammatory cell influx, 1 represents rare inflammatory cell influx (mild damage), 2 represents dense inflammatory cells in alveoli with preserved alveolar walls (moderate damage), and 3 represents dense inflammatory cells with undefined alveolar walls (severe damage).

### RNA sequencing

*S. salivarius* and *P. aeruginosa* were cultured individually and dually in 6-well plates in either TSBYE supplemented with 1% sucrose or in SCFM2 at 37 °C in 5% CO_2_ for 6 hours. Adherent biofilm cells were washed twice with PBS then collected for RNA isolation. RNA was isolated using the DirectZol RNA Mini Prep Kit (Zymo Research). mRNA sequencing was performed by the UAB Center for Clinical and Translational Science using an Illumina NextSeq 500 as described by the manufacturer (Illumina, Inc.). RNA Sequencing data have been deposited in NCBI’s Sequence Read Archive and are accessible through the BioProject accession number PRJNA771386.

### Glucose assay

*P. aeruginosa* and *S. salivarius* were subcultured to OD_600_ 0.5. 100μL of *P. aeruginosa* was inoculated into the bottom of a 6-well transwell (0.4μM, Corning) containing SCFM2. For dual species samples, 50μL of *S. salivarius* was inoculated into the top transwell insert containing SCFM2. Cultures were incubated for 6 hours at 37 °C with 5% CO_2_. *P. aeruginosa* cells were then collected from the bottom compartment of the transwell, pelleted, and lysed via glass bead beating. Intracellular glucose levels were measured via glucose assay (Abcam).

### Statistical analysis

All graphs represent sample means ± SEM. The Shapiro-Wilk normality test was used to determine distribution of datasets. Statistical analysis of normally distributed data was performed using either Student’s *t* test or one-way ANOVA with Šίdák’s multiple comparisons test. For nonparametric data, Kruskal Wallis with Dunn’s multiple comparisons test was used. Tests were performed using GraphPad Prism version 9 for Windows, La Jolla California USA, www.graphpad.com. Data were considered statistically significant if *p* < 0.05.

## Data availability statement

The datasets presented in this study can be found in online repositories. The names of the repository/repositories and accession number(s) can be found below: https://www.ncbi.nlm.nih.gov/, PRJNA771386.

## Ethics statement

The animal study was reviewed and approved by University of Alabama at Birmingham Institutional Animal Care and Use Committees.

## Author contributions

SS and JS designed the study. SS, JS, and JB performed experiments and conducted subsequent data analysis. LN performed histopathological analysis of tissue sections. SS and JS wrote the manuscript. All authors contributed to the article and approved the submitted version.

## Supplementary material

Supplementary Figure 1 IL-10 and IFN-γ responses during *S. salivarius* and *P. aeruginosa* single and dual infections. Production of cytokines **(A)** IL-10 and **(B)** IFN-γ in rats inoculated with Ss, PAO1, both, or sterile PBS.
