# Supplementary figures and images for: Commensal colonization reduces Pseudomonas aeruginosa burden and subsequent airway damage
Source: Front Cell Infect Microbiol. 2023 May 25;13:1144157. doi: 10.3389/fcimb.2023.1144157 (PMC10248150; doi:10.3389/fcimb.2023.1144157)

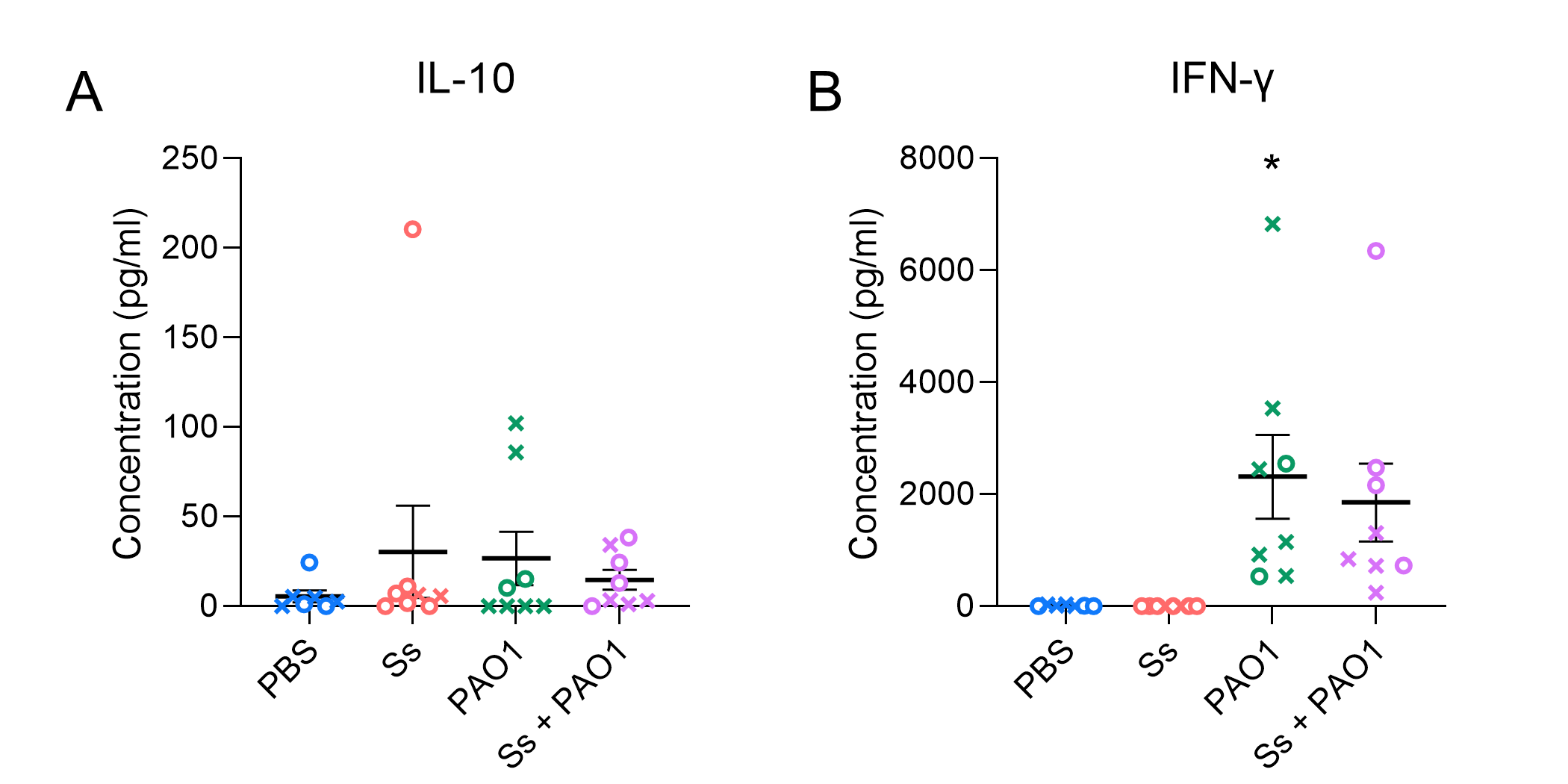

Supplement: Supplementary Figure 1 — IL-10 and IFN-γ responses during S. salivarius and P. aeruginosa single and dual infections. Production of cytokines (A) IL-10 and (B) IFN-γ in rats inoculated with Ss, PAO1, both, or sterile PBS. [file Image_1.tif]
